# Development and Validation of the Online Social Support for Smokers Scale

**DOI:** 10.2196/jmir.1801

**Published:** 2011-09-28

**Authors:** Amanda L Graham, George D Papandonatos, Hakmook Kang, Jose L Moreno, David B Abrams

**Affiliations:** ^1^Schroeder Institute for Tobacco Research and Policy StudiesAmerican Legacy FoundationWashington, DCUnited States; ^2^Cancer Control ProgramGeorgetown University Medical Center/Lombardi Comprehensive Cancer CenterWashington, DCUnited States; ^3^Center for Statistical SciencesBrown UniversityProvidence, RIUnited States; ^4^The Johns Hopkins Bloomberg School of Public HealthDepartment of Health, Behavior and SocietyBaltimore, MDUnited States

**Keywords:** Psychometrics, social support, Internet, smoking cessation

## Abstract

**Background:**

Social networks play an important role in smoking. Provision of social support during cessation is a cornerstone of treatment. Online social networks for cessation are ubiquitous and represent a promising modality for smokers to receive and provide the support necessary for cessation. There are no existing measures specific to online social support for smoking cessation.

**Objective:**

The objective was to develop a measure of social support to be used in online smoking cessation treatment research.

**Methods:**

Initial items for the Online Social Support for Smokers Scale (OS4) were based on existing theory and scales delineated in various taxonomies. Preliminary field analysis (N = 73) was conducted on 23 initial items to optimize the scale. Further development was conducted on a refined 15-item scale in the context of a large randomized trial of Internet and telephone cessation treatment with follow-ups at 3, 6, 12, and 18 months. In all, 1326 participants were randomized to an enhanced Internet arm that included a large online social network; psychometric analyses employed 3-month follow-up data from those reporting use of the enhanced Internet intervention at least once (n = 873). Items were subjected to a factor analysis, and the internal consistency reliability of the scale was examined along with construct and criterion validity. Other measures used in the study included demographics, nicotine dependence, partner support for cessation, general social support, social integration, stress, depression, health status, online community use, Internet use behaviors, intervention satisfaction, and 30-day point prevalence abstinence.

**Results:**

The final 12-item OS4 scale demonstrated high internal consistency reliability (Cronbach alphas .86-.89) across demographic and smoking strata of interest. The OS4 also demonstrated good construct and criterion validity, with the directionality of the observed associations providing support for most a priori hypotheses. Significant Pearson correlations were observed between the OS4 and the Partner Interaction Questionnaire (PIQ) Positive subscale (ρ = .24, *P* < .001). As hypothesized, participants with the highest OS4 scores were more likely to have actively participated in the enhanced Internet community and to have high levels of satisfaction with the enhanced Internet intervention. In logistic regression analyses, the OS4 was highly predictive of 30-day point-prevalence abstinence at 6, 12, and 18 months (all *P* values <.001). The odds of abstinence at 6 months rose by 48% for each standard unit increase in online social support (95% confidence interval [CI] 1.17 - 1.71), dropping only slightly to 37% at 12 and 18 months (95% CI 1.17 - 1.59).

**Conclusions:**

The OS4 is a brief, reliable, and valid instrument for measuring online social support for smoking cessation. Results should be replicated and extended, but this study suggests the OS4 can be used to advance theory, understand mechanisms, and potentially help to improve the tailoring of Internet-based smoking cessation treatments. It can also inspire development of similar measures for other online health-related intervention research.

**Trial registration:**

Clinicaltrials.gov #NCT00282009; http://clinicaltrials.gov/ct2/show/NCT00282009 (Archived by WebCite at http://www.webcitation.org/60XNj3xM6)

## Introduction

It has long been recognized that social support and social connections play an important role in smoking initiation, maintenance, quitting, and relapse. High levels of social support have been linked to better cessation outcomes in correlational and epidemiological studies [1-4] and low levels of support (eg, negative behaviors from a spouse) are a barrier to cessation and maintenance [2,4,5]. Recent analyses by Christakis and colleagues [6] demonstrated that social networks play a powerful role in smoking cessation in that cessation propagates more rapidly among smokers in the proximal social network of a quitter. Given the available evidence, the provision of social support during the process of quitting is one of the cornerstones of evidence-based cessation treatment [7].

Online social networks for smoking cessation have become ubiquitous and, thus, may represent a promising modality for smokers to both receive and provide the kind of support necessary for cessation and relapse prevention. Through Internet-based social networks, smokers have round-the-clock access to thousands of other individuals who are actively quitting smoking, struggling to maintain abstinence, or celebrating various milestones of abstinence. Access in real time to a diverse mix of individuals in all stages of the cessation journey is a unique aspect of online social networks. No other cessation treatment modality provides an ongoing opportunity for current and former smokers to interact and influence each other. In addition, smokers benefit not only from active interactions with other network members, but also from various passive sources of social influence and social support. Smokers can establish personal connections with other network members, or can browse (“lurk”) the messages and profiles of others. These kinds of active and passive interactions may influence an individual’s motivation to quit, reinforce the undesirability of smoking, assist in buffering cessation-related stressors, enhance coping skills, and provide suggestions for eliminating smoking cues.

To date there have been few published studies of online social networks for cessation. Several studies have described the frequency, intensity, and nature of interactions among online social network members [8-12]. Other studies have examined the association of participation in online communities with cessation outcomes [11,13,14]. These associations appear to be relatively robust, with higher levels of social exchanges (eg, messages, forum posts, and blog posts) and social connectedness (eg, number of buddies and number of people sending messages to and receiving messages from) associated with higher likelihood of abstinence. While these associations are compelling, we know of no studies that have examined whether interactions in an online social network for cessation do, in fact, lead to changes in perceived social support. If observational findings are to be harnessed in interventions that attempt to manipulate social support to improve cessation outcomes, a measure of perceived social support from online social networks is needed, both as a manipulation check and also as a measure of a potentially important mediating mechanism. To our knowledge, there are no existing measures of online social support specific to smoking cessation.

Thus, the purpose of the present study was to develop a brief, reliable, and valid measure of social support for online smoking cessation research that could be used with minimal burden on respondents. Following item generation, we conducted a series of psychometric analyses to examine the performance of individual items and to optimize the scale. We subjected items to a factor analysis and examined the quality of the scale as reflected by the internal consistency reliability coefficient, Cronbach alpha. Finally, we examined the construct and criterion validity of the scale, relying on published studies to guide our hypotheses where possible.

Construct validity is the extent to which a psychometric scale, as operationalized in a particular study, actually measures the theoretical construct that it purports to measure. This requires evidence of similarity with measures known to be related to the underlying construct (convergent validity), and lack of similarity with measures of theoretically distinct constructs (discriminant validity) [15]. With regard to convergent validity, we hypothesized that the scale would be positively correlated with other measures of social support, including smoking-specific social support from a partner or friend [2,16], a general multidimensional measure of support [17], and measures of social integration [18] and of frequency of online communications via social media. With regard to discriminant validity, we hypothesized that our measure of online perceived support would show no association with smoking variables (nicotine dependence, age of first smoking, daily smoking rate, number of quit attempts in the past year, desire to quit, or confidence in quitting), psychosocial variables (stress, current depressive symptoms, and history of anxiety/depression), perceived health status, or duration and frequency of Internet use.

Criterion validity examines the degree to which test scores on a particular psychometric scale correlate well with one or more criteria taken as representative of the construct. This requires evidence of similarity with criteria obtained at approximately the same time (concurrent validity), as well as following test administration (predictive validity) [15]. With regard to concurrent validity, we hypothesized that higher scores on our measure of online social support would distinguish those with higher levels of satisfaction with the website; those who participated in the online community more intensively as indicated by self-report data (amount of perceived “help” given to and received from other community members); and those who participated in the online community more intensively as indicated by automated tracking of online activities (ie, use of any community features, internal messages sent to other members, and buddies in the online community). With regard to predictive validity, we hypothesized that participants with higher scores of online social support 3 months after beginning their participation in an online cessation intervention would be more likely to be abstinent at each of the subsequent follow-up assessments.

## Methods

### Overview

Development of the Online Social Support for Smokers Scale (OS4) was conducted in the context of a randomized controlled trial (Clinicaltrials.gov #NCT00282009) of Internet cessation treatment alone and in conjunction with proactive telephone counseling [19,20]. We developed the OS4 in order to examine theory-driven hypotheses about the role of online social support as a potential mediating mechanism of treatment outcome. Briefly, the trial randomized 2005 current smokers to one of three treatment conditions: (1) enhanced Internet (n = 651), (2) enhanced Internet plus proactive telephone counseling (n = 675), or (3) a static, information only basic Internet comparison condition (n = 679). Participants were followed at 3, 6, 12, and 18 months postrandomization.

For the enhanced Internet condition, participants were provided free access to QuitNet.com, an interactive, commercial cessation website that provides evidence-based cessation treatment in accordance with national guidelines [7]. Described elsewhere [11,19], QuitNet provides (1) advice to quit; (2) assistance in setting a quit date; (3) assessment of motivation, smoking history, demographics, and nicotine dependence; (4) individually-tailored information; (5) problem solving/skills training content; (6) tailored assistance in using pharmacotherapies approved by the US Food and Drug Administration; and (7) social support within its large online social network [10]. For over 10 years, QuitNet has enrolled individuals into a network of current and former smokers seeking to quit or stay quit and provided multiple mechanisms of social support and social influence. QuitNet’s community features allow for multiple forms of social support and social influence. Communication can occur through asynchronous channels, such as private internal email (“Q-Mail”) or one-to-many messaging in the threaded forums, as well as synchronous channels such as chat rooms. Users can self-affiliate into clubs that are essentially user-initiated mini-sites complete with a dedicated forum. “Buddy lists” allow individuals to keep track of their friends. Social influence regarding cessation is conveyed through profile pages, journals (similar to a blog), anniversary lists, and testimonials. Users are encouraged to publically share their quit dates, which are set through a “wizard” tool, and users are prompted for updates at each login. QuitNet maintains a complete transactional history of all events, including communications that occur throughout the site. Active events (eg, sending internal email or posting a public message) and passive actions (eg, reading messages or viewing another individual’s profile) are logged into a relational database.

### Questionnaire Development

Development of the OS4 began with a comprehensive review of the literature to gather existing measures of social support specific to smoking cessation and more broadly related to health behavior change. We also gathered unpublished items being used in ongoing studies from tobacco experts. Measures of social support often distinguish between socially supportive functions that are perceived to be available (*perceived support*) and functions that were recently provided (*received support*) [21]. Given reports that a small percentage of people actively participate in online networks for cessation [11,13,14] and that a much larger number of people browse/lurk in the community, we included items that addressed both perceived and received support in order to account for the possibility that the potential availability of support as needed is as important as support actually received.

Initial items were based on existing scales but were adapted to reflect the specific social context of Internet interventions. Items covered each of the five domains of social support delineated in various taxonomies [22-24]: (1) emotional or esteem support, which refers to the availability of people to talk to about one’s problems who can provide indications of caring and acceptance, empathy, reassurance, liking, and respect; (2) instrumental or tangible support, which refers to the perceived availability of material aid or practical support; (3) informational support, which refers to advice or guidance to solve a problem; (4) companionship or belonging support, which refers to perceived social companionship or social integration; and (5) validation or appraisal support, which provides feedback or social comparison about the normative nature of an individual’s behaviors or feelings and their relative status in the population. A total of 23 items were generated to provide adequate redundancy within each domain. Items were written at a sixth grade reading level.

Preliminary field testing of the OS4 items was conducted within the QuitNet online social network. Using the internal QuitNet mail system, an invitation to complete an online survey was sent to active community members who had logged into the system at least 10 times. This criterion was selected to ensure that respondents had adequate experience within the community to knowledgeably respond to the relevance or appropriateness of the items. Participants were asked to respond to each item using a 5-point scale where 1 = definitely false, 2 = probably false, 3 = no opinion, 4 = probably true, and 5 = definitely true. Next to each item, participants could also enter comments about the relevance or appropriateness of the item or suggest alternate wording.

A total of 85 people visited the survey link between June 6, 2005, and June 10, 2005; of these, 73 completed the survey. We examined the mean, standard deviation, and range of the original 23 items as well as feedback provided about specific items. A total of 13 items with little variability and/or wording that participants indicated was unclear or tangential to their experience in the community were dropped. Based on participant feedback and expert review, modifications were made to the 10 remaining items to enhance their clarity and maximize their relevance, and 5 new items were added. As shown in Table 1, the scale was composed of 15 items (3 items in each of the 5 domains mentioned previously).

**Table 1:** Online Social Support Scale for Smokers: Original scale items^a^

Q1. I connected with other people on QuitNet on topics other than smoking.
Q2. I never posted messages on QuitNet.^b^
Q3. I felt comfortable sharing private or personal thoughts with other members of QuitNet in the public forums.
Q4. I felt comfortable sharing private or personal thoughts through Q-Mail to individual members of QuitNet.
Q5. By giving advice to other members of QuitNet, my own efforts in quitting were reinforced.
Q6. Being anonymous made it easier to share personal information with people on QuitNet.
Q7. Using QuitNet helped me cope with cravings.
Q8. I got advice and support on QuitNet that I could not find anywhere else.
Q9. It was comforting to know that I wasn’t alone in the struggle to get and stay quit.
Q10. The fact that QuitNet is available whenever I need it, night or day, was important to me.
Q11. I felt supported and encouraged by other QuitNet members.
Q12. Advice and support from people in different stages of quitting was helpful to me.
Q13. I received negative or critical comments from other QuitNet members.^b^
Q14. I received some bad information or advice from someone on QuitNet.^b^
Q15. Being in a different time zone from other members made it difficult to get the support I needed.^b^

^a^ Scoring structure: 1 = disagree a lot, 2 = disagree a little, 3 = agree a little, 4 = agree a lot

^b^ reverse-scored item

### Procedure

At each follow-up assessment, participants randomized to the enhanced Internet and enhanced Internet plus telephone treatment arms were asked how many times they had used the QuitNet website during the follow-up period. Those who had used the website at least once were administered the refined 15-item OS4. The psychometric analyses reported here used data from the 3-month follow-up, since website utilization is highest immediately after registration and tails off within 3 months for the majority of new members. Of the 990 participants who were reached at the 3-month follow-up, 873 participants reported using the QuitNet website at least once and completed the OS4; these participants were used as a validation sample. The remaining 117 participants reported no use of the website during the first 3 months of the study; this sample was used to examine generalizability.

### Measures

#### Demographics and Smoking History

Demographic information collected at baseline included age, gender, education, race, ethnicity, household income, marital status, and employment status. The smoking history questionnaire assessed age of first smoking, daily smoking rate, and number of intentional quit attempts in the past year. Desire and confidence in quitting were each measured on a scale of 1 to 10 where 1 = not at all and 10 = very much.

#### Nicotine Dependence

Nicotine dependence was measured using the Fagerstrom Test for Nicotine Dependence (FTND) [25], a 6-item measure of dependence considered a standard instrument in the field. Greater scores indicate higher levels of dependence. Internal consistency reliability at 3 months was moderate (Cronbach alpha = .69).

#### Partner Support for Cessation

The Partner Interaction Questionnaire (PIQ) [2] is the most commonly used measure of spouse/partner support related to cessation. We administered a modified version of the PIQ that measures the receipt of specific behaviors from the person who follows a participant’s efforts to quit smoking most closely, not just a spouse/partner [26,27]. The modified version used a 5-point Likert scale to assess how frequently the participant’s support person exhibited 3 positive and 3 negative behaviors [16]. Positive items were “express pleasure at your efforts to quit,” “congratulate you for your decision to quit smoking,” and “express confidence in your ability to quit/remain quit.” Negative items were “mention being bothered by smoke,” “ask you to quit smoking,” and “criticize your smoking.” Response options were 0 = never, 1 = almost never, 2 = sometimes, 3 = fairly often, and 4 = very often. For the PIQ scale, internal consistency reliability at 3 months reached .84 for the Positive subscale, .85 for the Negative subscale, and .74 for the difference of the two.

#### General Social Support

The 12-item version of the Interpersonal Support Evaluation List (ISEL) [17] was used to assess the perceived availability of social resources. The ISEL is composed of three subscales that assess the perceived availability of distinct functions of social support: the Appraisal subscale measures the perceived availability of someone to talk to about one’s problems; the Belonging subscale assesses the perceived availability of people with whom to engage in activities; the Tangible subscale measures the perceived availability of instrumental support or material aid. A total measure of perceived support can also be calculated. Respondents indicated their level of agreement with each statement using a 4-point scale: 0 = disagree a lot, 1 = disagree a little, 2 = agree a little, and 3 = agree a lot. At the 3-month follow-up, internal consistency reliability reached .82 for Appraisal, .76 for Belonging, .67 for Tangible, and .87 for the overall scale.

#### Social Integration

Designed as a measure of social integration, the Social Network Index [18] assesses participation in twelve types of social relationships (eg, spouse, friend, workmate, volunteer). Social network diversity is calculated by assigning one point for each type of relationship (possible score of 12) for which respondents indicate communication at least every 2 weeks. The total number of network members is calculated as the total number of persons with whom they speak at least once every 2 weeks.

#### Perceived Stress

The 4-item Perceived Stress Scale (PSS) [28] assesses the degree to which participants find their lives to be unpredictable and uncontrollable. Each item is rated on a 5-point Likert scale to indicate how frequently the individual has felt a particular way during the past month. Response options were 0 = never, 1 = almost never, 2 = sometimes, 3 = fairly often, and 4 = very often. Internal consistency reliability was .82 at 3 months.

#### Depression

Symptoms of current depression were measured using the 10-item Center for Epidemiological Studies Depression Scale (CES-D) [29]. Participants indicated the frequency of occurrence of each symptom during the past week (less than a day, 1-2 days, 3-4 days, and 5-7 days). Internal consistency reliability was .82 at 3 months. Participants also reported past year diagnosis of nervous trouble or depression (yes/no).

#### Perceived Health Status

Using the item from the Medical Outcomes Study 36-Item Short-Form Health Survey (SF-36), participants rated their current health status on a 5-point scale from 1 (excellent) to 5 (poor) [30].

#### Internet Use Behavior

Participants reported the number of years they had used the Internet and the frequency of Internet use. Participants also rated the frequency of online communications other than email (eg, blogging and use of chat rooms): 1 = never, 2 = less often, 3 = every few weeks, 4 = 1 to 2 days a week, 5 = 3 to 5 days a week, 6 = about once a day, 7 = several times a day.

#### Intervention Satisfaction

At each follow-up, participants rated their overall satisfaction with the QuitNet website, its perceived helpfulness, and how well the website met their expectations on a scale from 1 to 10 where 1 = not at all and 10 = very much. In addition, participants indicated how much help they had provided to other QuitNet community members and how much help they had received from other QuitNet community members on a 4-point scale where 1 = none, 2 = a little, 3 = some, and 4 = a lot.

#### Online Community Participation Metrics

At the 3-month follow-up, the following selected metrics of active participation in the QuitNet community were extracted: (1) any use of community features; (2) number of Q-Mails sent to other members; and (3) number of buddies designated. These particular metrics were selected based on their expected association with a measure of perceived support.

#### Smoking Cessation

At each follow-up assessment, participants self-reported smoking status over the past 30 days which was used as the primary outcome for the parent trial [20].

### Data Analysis

Data analysis was conducted in multiple phases. First, to examine generalizability, we compared the validation sample to the 117 participants who had not used the website on a range of baseline demographic, smoking, and psychosocial variables. Two-sample *t* tests were used for continuous variables, Chi-square tests were used for categorical variables, and Poisson regression was used for count data. For categorical variables with small cell frequencies (<5 subjects per cell), significance levels were computed using Fisher’s exact test. Sensitivity of *t* test findings to skewness in continuous variables was assessed by Wilcoxon signed-rank tests.

Next, we performed a factor analysis on the OS4 of the interitem covariance matrix using maximum likelihood estimation followed by a varimax rotation. The loadings from the resulting 2-factor solution were used to construct a low-dimensional representation of the interitem covariance matrix known as a biplot [31,32]. By representing each item by a directed arrow, biplots can be used to visually examine individual item characteristics and between-item relationships in cases where the number of items is too large to allow such information to be easily discerned from the interitem covariance matrix itself. Arrow orientation depends on item loadings, with arrows in the first quadrant having positive loadings on both the first and second factors. Arrows pointing in the same direction indicate items that are positively correlated; arrows pointing in the opposite direction indicate negatively correlated items; uncorrelated items have arrows that appear at right angles. Arrow length is related to item variability, with long arrows reflecting highly variable and, hence, more informative items. Long arrows that overlap are indicative of items that may be informative on their own but potentially contribute redundant information. Short arrows are indicative of items with little between-subject variability in the space of the first two factors, the implication being that they can be dropped from the measurement scale with little loss of information about the underlying construct. A biplot suggestive of a single factor solution would be one in which all arrows lie in the positive quadrant after rotation. When the arrows not only point in the same direction but are also approximately equal in length, then all items are informative to a similar degree and a total score should provide a good approximation to the factor score from a single factor solution.

After dropping 3 uninformative items, we repeated the factor analysis, comparing the 1-factor and 2-factor solutions in terms of proportion of variance explained among the remaining 12 items. Once a single-factor solution was established, we used Cronbach alpha to determine whether deletion of partially overlapping items would adversely affect overall scale reliability, with a value of .80 set as the lower acceptable bound [33]. To ensure that use of the OS4 is appropriate for various subgroups of smokers, we examined changes in reliability across population strata defined by gender, race/ethnicity (non-Hispanic white vs other categories of race/ethnicity), marital status, education, and income. Finally, we examined the correlation between the actual factor scores and the total score obtained by simply adding the items loading on a particular factor. When the two scores are highly correlated, there is little information lost when calculating subject-specific measures of online social support for quitting smoking by weighing each scale item equally instead of using the optimal item weights suggested by the factor analysis itself.

Having finalized our choice of weights for the items used to measure online social support, we proceeded to examine construct validity. Convergent validity was assessed using cross-sectional associations between the OS4 and other theory-driven measures of social support. Discriminant validity was assessed using cross-sectional associations between the OS4 and theoretically distinct measures (ie, smoking variables, stress and depression, perceived health status, and Internet use variables). The association between variables of interest and OS4 was measured using Pearson correlations for continuous variables and polyserial correlations for binary variables. Correlations were corrected for attenuation due to measurement error by inflating them by the inverse square root of the reliability coefficients of the respective psychometric scales in our validation sample. In addition, significance levels were corrected for finite sample uncertainty in estimation of the reliability coefficients themselves [34]. No correction was made for the lack of perfect reliability in the OS4 itself, as one must use the OS4 scale as it exists rather than in terms of its unknown “true score” [35].

To assess concurrent criterion validity, we compared the three metrics of intervention satisfaction (overall satisfaction, perceived helpfulness, the degree to which the site met expectations) and metrics of active participation in the community (any community use, sent any Q-Mail, designated any buddies, and gave or received some or a lot of help to or from other members) at 3 months across OS4 quartiles using normal linear regression for continuous variables and logistic regression for binary variables. Statistically significant between-group differences in means or proportions were taken as evidence that the OS4 does indeed have the discriminatory power one would have expected it to demonstrate on such measures. Finally, predictive criterion validity was assessed in terms of the ability of the OS4 at 3 months to predict self-reported 30-day point prevalence abstinence at 6, 12, and 18 months in a logistic regression model that controlled for the effects of treatment assignment (enhanced Internet vs enhanced Internet + phone).

## Results

### Participants

Detailed characteristics of all trial participants have been reported elsewhere [20]: There were no differences on any of the demographic, smoking, or psychosocial variables across treatment arms. Briefly, among the 873 participants in the validation sample, mean age was 36.5 years (SD 11.1) and 52.2% (456) were female. Most were white (786 or 88%), had completed at least some college (688 or 79%), were employed full-time (605 or 69.3%), and were long-term and frequent Internet users: 81.4% (709) had used the Internet for more than 5 years, and 79.4% (693) used the Internet several times a day. Participants smoked an average of 20 cigarettes per day (SD 9.4), reported a high level of desire to quit (mean 9.04, SD 1.3) and slightly lower confidence in quitting (mean 6.26, SD 2.2), and had made an average of 3.4 (SD 10.2) quit attempts in the past year.

To examine generalizability, we compared the validation sample with the 117 participants who did not use the enhanced Internet treatment during the first 3 months of the study. The validation sample had a higher percentage of women (52.2% or 456/873 vs 41.0% or 48/117, *P* = .02), reported lower levels of nicotine dependence (mean 5.1, SD 2.3 vs mean 4.5, SD 2.5, *P* = .02), and was more likely to use the Internet on a daily basis to communicate via blogs or instant messaging (40.8% or 356/873 vs 33.3% or 39/117, *P* = .009). There were no differences on any of the other variables examined.

### Factor Analysis of the Covariance Matrix

**Figure 1:**
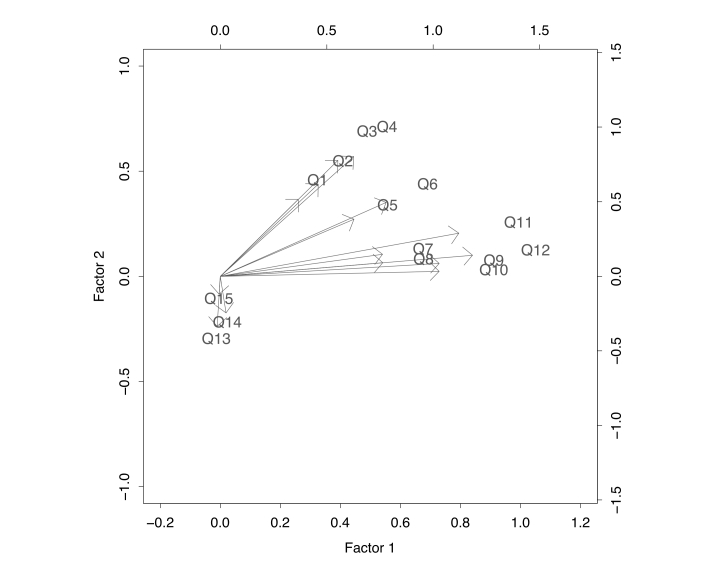
Biplot of Covariance Matrix.

Results from the factor analysis of the items in Table 1 are presented in Figure 1 in biplot form (see Multimedia Appendix 1 for interitem correlation matrix). Factor analysis of the 15 items revealed a homogeneous cluster of 12 items (Q1 through Q12) with strong intraclass correlation (ICC = .36) and high reliability (Cronbach alpha = .87). The 3 remaining items (Q13 through Q15) appeared relatively uninformative. Further examination of their frequency distribution indicated that Q13 and Q14 showed minimal between-subject variation, with 84% and 90% of the sample respectively endorsing the highest (ie, least negative) category. In contrast, Q15 showed larger between-subject variation, but seemed to lie in a dimension other than that described by the first two factors depicted in Figure 1. Therefore, we decided to drop these 3 items from further consideration.

The biplot also suggested that the remaining items could be grouped into 2 highly correlated subsets (Q1 through Q4 and Q7 through Q12), with Q5 and Q6 equidistant from them. Examination of factor loadings from a 2–factor solution of Q1 through Q12 indicated that Q7 through Q12 defined a single factor explaining 23.6% of the variance, while Q1 through Q4 defined a second factor explaining 21.5% of the variance; Q5 and Q6 had approximately equal loadings on each of these factors. However, any increases in intraclass correlation among the items of these two possible online support subscales could not compensate for decreases in subscale-specific reliability due to halving the number of items loading on each (Cronbach alpha < .80 for both). Therefore, we decided to treat all 12 items as belonging to a single construct, with the resulting 1-factor solution explaining 37.3% of the variance versus a combined 45.1% for the 2-factor solution. 

Stratification by gender, race/ethnicity (non-Hispanic white vs other), marital status, education, and income showed that reliability remained high across all subgroups of interest (Cronbach alpha .86-.89).

As expected by the length of the arrows in Figure 1, the 1-factor solution was dominated by highly informative items Q11 and Q12. However, the magnitude of the loadings showed only moderate variation across items, ranging from .44 to .78. This led us to consider the possibility of using the total score of Q1 through Q12 as a scalar summary of online social support for quitting smoking. The resulting Pearson correlation (ρ = .99) between the total and factor scores suggested that the two measures were extremely highly correlated. Given the simplicity of calculating the Q1 through Q12 total score, we decided to use it as a proxy of the factor score from a single factor solution.

### Construct Validity

Table 2 presents results of convergent validity analyses. After correction for attenuation, Pearson correlations in the moderate range (ρ = .10-.30) according to Cohen’s definition [36] were observed between the OS4 and the PIQ Positive subscale (ρ = .24, *P* < .001), and between the OS4 and the difference between the PIQ Positive and Negative subscales (ρ = .20, *P* < .001). Despite the large sample size (n = 873), which was large enough to guarantee 84% power to detect Pearson correlations with magnitude as low as .10, the correlation between the OS4 and the Negative subscale of the PIQ failed to attain significance. Unexpectedly, small correlations were also detected with the ISEL Total Score (ρ = .06, *P* = .08) and the Appraisal (ρ= .07, *P* = .05) and Belonging (ρ=.08, *P* = .04) subscales; the correlation of the OS4 with the ISEL Tangible subscale was not significant. As expected, correlations between the OS4 and metrics of social network integration (number network members: ρ = .08, *P* = .02; social network diversity: ρ = .16, *P* < .001) as well as with frequency of Internet communications via social media (ρ = .16, *P* < .001) were in the positive direction but fell in the small-to-moderate range.

As hypothesized with regard to discriminant validity, results presented in Table 2 suggest that there was no association of the OS4 with the FTND Total Score, age of becoming a daily smoker, the number quit attempts in the past year, current symptoms of stress and depression, perceived health status, and duration and frequency of Internet use. Small-to-moderate correlations were observed between the OS4 and daily smoking rate at baseline (ρ = -.15, *P* < .001), desire to quit (ρ = .12, *P* = .006), confidence in quitting (ρ = .09, *P* = .04), and a past-year diagnosis of anxiety/depression (polyserial ρ = .12, *P* = .01).

**Table 2:** Construct validity analyses

			Correlation With OS4 Total Score	Correlation^a^ With OS4 Total Score	*P* value^a^
**Convergent validity**					
	**Partner Interaction Questionnaire (PIQ)**				
		Positive subscale	.224	.244	<.001
		Negative subscale	-.033	-.035	.36
		Positive-Negative subscale	.168	.195	<.001
	**Interpersonal Support Evaluation List (ISEL)**				
		Appraisal subscale	.066	.073	.053
		Belonging subscale	.069	.079	.04
		Tangible subscale	.012	.015	.73
		Total Score	.060	.064	.08
	**Social Network Index (SNI)**				
		Number of network members	.077		.02
		Social network diversity	.159		<.001
		Frequency of Internet Communications	.156		<.001
**Discriminant validity**					
	**Smoking variables**				
		Fagerstrom Test for Nicotine Dependence (FTND) total score	-.045	-.054	.32
		Age of becoming a daily smoker	.000		.99
		Daily smoking rate (at baseline)	-.147		<.001
		Number quit attempts past year	-.039		.28
		Desire to quit	.122		.006
		Confidence in quitting	.092		.04
	**Psychosocial variables**				
		Perceived Stress Scale (PSS)	-.020	-.022	.58
		Center for Epidemiological Studies Depression Scale (CES-D)	-.009	-.010	.79
		Past year diagnosis anxiety/depression (yes/no)	.116		.01
	**Health status**				
		Perceived health status	-.049		.17
	**Internet use**				
		Duration of Internet use	-.035		.24
		Frequency of Internet use	-.042		.32

^a^ After correction for attenuation due to measurement error

### Criterion Validity

In order to examine concurrent criterion validity, we first standardized the observed OS4 total score (mean 31.44, SD 7.96) to zero mean and unit variance in our overall sample and then calculated its average value within quartiles of the criterion of interest. As a result, observed between-group differences can be directly compared with Cohen’s definitions [36] of effect size for continuous outcomes (small = .20, moderate = .50, large = .80), providing a yardstick for the practical significance of the findings.

Table 3 shows that higher ratings on each of the variables measuring satisfaction with the enhanced Internet website were associated with higher total scores on the OS4. Further, pairwise differences in mean OS4 total score across successive quartiles of intervention satisfaction level were all statistically significant (all *P*’s < .004), irrespective of the quartiles of interest and of the particular metric used to measure satisfaction levels. Discriminatory power was strong across the entire range of intervention satisfaction, with large differences in mean OS4 total score at the lower end of intervention satisfaction and moderate differences elsewhere (quartile differences: 1st vs 2nd = .64-.84, 2nd vs 3rd = .27-.44, 3rd vs 4th = .33-.53).

**Table 3:** Average OS4 score by level of intervention satisfaction

	Quartile of Intervention Satisfaction Metric			
	1st	2nd	3rd	4th
Satisfaction with website	-0.82	-0.18	0.20	0.53
Website met expectations	-0.78	0.10	0.17	0.51
Perceived helpfulness	-0.95	-0.19	0.22	0.75

Similarly, Table 4 shows that active participants in the online community had significantly larger OS4 scores than nonparticipants (all *P*’s < .001) on all of the participation metrics (use of any community features, internal messages sent to other members, and online buddies).

**Table 4:** Average OS4 score by community participation

	No	Yes	Difference
Used community features	-0.42	0.27	0.69
Sent Q-Mail	-0.24	0.93	1.17
Acquired online buddies	-0.08	1.44	1.52

With regard to predictive validity, logistic regression analyses revealed that 3-month values of the OS4 (mean 31.44, SD 7.96) adjusted for differences between treatment arm (enhanced Internet vs enhanced Internet + phone) were highly predictive of 30-day point-prevalence abstinence at 6, 12, and 18 months (all *P*’s < .001). Among those reached at follow-up, the odds of abstinence at 6 months rose by 48% for each standard unit increase in OS4 total score (adjusted odds ratio [OR] = 1.48, 95% CI 1.17-1.71), dropping only slightly to 37% at 12 and 18 months (adjusted OR = 1.37, 95% CI 1.17-1.59).

## Discussion

The OS4 is a specific measure of online social support for smoking cessation, developed using previous theory and measures that have shown promise in understanding the role of social support in smoking cessation and relapse prevention. Developed within the context of a treatment outcome study of Internet and telephone treatment for smoking cessation, the OS4 emerged as a reliable and valid instrument. In a relatively large sample of Internet users, this 12-item scale demonstrated strong intraclass correlations across sociodemographic groups, resulting in high internal consistency reliability (Cronbach alpha .86-.89).

The OS4 demonstrated adequate construct validity. With regard to convergent validity, small-to-moderate correlations were observed with the Positive Support subscale of the PIQ, but not with the Negative Support subscale, a discrepancy that may be due to the fact that the OS4 was designed to capture the positive elements of supportive interactions in an online social network for cessation. A positive correlation with the ISEL Total Score was smaller than expected due to a lack of correlation between the OS4 and the Tangible subscale which contained items largely irrelevant to the experience of individuals interacting in an online social network (eg, If I was stranded 10 miles from home, there is someone I could call who could come and get me; If I were sick, I could easily find someone to help me with my daily chores). Small-to-moderate correlations were also observed with measures of social integration and the frequency of online communications via blogs and chat rooms. The OS4 also showed good discriminant validity in that it was not associated with measures such as nicotine dependence, general Internet use, and health status. Significant relationships of the OS4 with daily smoking rate, desire and confidence in quitting, and a history of nervous trouble/depression were quite small in magnitude but may indicate that smokers who are more motivated or who perceive the need for greater assistance in quitting tend to proactively reach out for support and engage in the community to a greater degree. Future research will need to clarify the nature of these associations.

All hypotheses regarding criterion validity were strongly supported. Concurrent validity was demonstrated by the significantly higher scores on the OS4 observed among subjects who reported higher levels of customer satisfaction as well as those who actively participated in the online community. Importantly for cessation research, the OS4 demonstrated excellent predictive validity with higher scores at 3 months predicting a greater likelihood of abstinence at 6, 12 and 18 months. Indeed, the OS4 may help to provide new insights into the role of social support in the cessation process and effective ways to harness support in intervention research. Despite historically robust associations between social support and better cessation treatment outcomes, numerous attempts to increase social support and enhance treatment effectiveness have been largely ineffective in increasing abstinence. For the most part, these interventions took place in face-to-face treatments and included spouse or partner training and “buddy” interventions [37-40]. Several explanations have been offered for the lack of effectiveness of these kinds of support interventions. First, it may be difficult to change longstanding interpersonal dynamics through interventions with a spouse or partner. Second, intensive face-to-face treatment programs may provide a sufficient level of support such that additional components provide no added value with respect to social support; the challenge, however, is that less than 5% of smokers are interested in attending face-to-face treatment programs [41], making it critical to identify an appealing and accessible treatment modality that can provide the same type of intensive support. Third, social support may be a stable or “traitlike” construct that is resistant to change within a time-limited intervention; traditional treatment programs are time-limited in nature, typically lasting only 8 to 12 weeks. It may be that the creation or modification of meaningful supportive relationships requires more sustained interaction than traditional modalities can provide. Finally, it may be that the number and/or characteristics of people in an individual’s “real-world” (ie, face-to-face) network may not be sufficient to provide the type or frequency of supportive interactions necessary to influence cessation outcomes.

The changing landscape of Internet-based social interactions and the ubiquity of online social networks provide an exciting opportunity to revisit social support mechanisms and interventions. By their nature, online social networks for cessation now make possible the provision and receipt of support in ways that were not feasible, convenient, or practical within face-to-face interventions or social networks of family and friends. The Internet affords continuous and real-time availability of thousands of supportive others in all stages of the quitting process, the rapid spread of information through network ties, and the ability to remain anonymous, among other factors. Christakis and colleagues [6,42] showed recently that smoking cessation and obesity spread more rapidly in the proximal social networks of probands than in unrelated networks, illustrating the importance of network effects in addition to interindividual effects in the social support process. As an assessment instrument specifically designed for exploring the links of perceived online social support to intermediate variables and cessation outcomes among those trying to quit, the OS4 may be helpful in advancing theory and improving the design and effectiveness of online cessation interventions.

Results should be considered in the context of several limitations. First, the measure was derived and validated on the same sample, potentially exaggerating the significance of the findings. Future work is needed to independently validate the factor structure, reliability, and validity of the OS4 in a new and different sample of smokers. Second, the measure was developed specifically within the context of one Web-based smoking cessation intervention, QuitNet.com. Development efforts ensured that items were relevant to the features and functionality of this particular website, and items specifically referenced QuitNet. Other research will need to adapt this measure to the specific Internet resource being evaluated and confirm that items remain relevant.

In summary, the 12-item OS4 is a reliable and valid instrument, developed to advance understanding of the emergent role of online social networks for smoking cessation treatment. To our knowledge, it represents the first psychometric scale developed for this purpose and is a relatively brief instrument that can be included in intervention research where response burden is a concern. The measure can also improve our understanding of basic mechanisms of action, develop and advance theories of behavior change on the Internet, and inform the development of tailored interventions to improve the effectiveness of interventions on cessation outcomes and relapse prevention. Development of an instrument to measure online social network and support activities, such as the OS4, can also inspire similar work in other areas of health promotion, disease prevention, and chronic disease management where social support also plays an important role.

## Multimedia Appendix 1

Inter-item correlation matrix of OS4.

## Abbreviations

CES-D: Center for Epidemiological Studies Depression Scale
FTND: Fagerstrom Test for Nicotine Dependence
ISEL: Interpersonal Support Evaluation List
OS4: Online Social Support for Smokers Scale
PIQ: Partner Interaction Questionnaire
PSS: Perceived Stress Scale
SNI: Social Network Index
