# Supplementary material for: Development and Validation of the Online Social Support for Smokers Scale
Source: J Med Internet Res. 2011 Sep 28;13(3):e69. doi: 10.2196/jmir.1801 (PMC3222172; doi:10.2196/jmir.1801)
Supplement: Supplementary file 1 [file jmir_v13i3e69_app1.pdf]

Multimedia Appendix 1. Inter-item correlation matrix of OS4

	Q2	Q3	Q4	Q5	Q6	Q7	Q8	Q9	Q10	Q11	Q12	QT
Q1	.31	.40	.41	.22	.33	.21	.20	.20	.20	.24	.29	.76
Q2		.46	.54	.41	.28	.28	.18	.23	.27	.25	.41	.74
Q3			.66	.48	.36	.27	.25	.33	.36	.39	.24	.72
Q4				.48	.46	.27	.25	.35	.38	.30	.33	.72
Q5					.38	.38	.30	.34	.27	.25	.40	.70
Q6						.21	.24	.30	.32	.35	.20	.62
Q7							.44	.32	.28	.18	.31	.62
Q8								.35	.41	.39	.20	.61
Q9									.21	.24	.22	.60
Q10										.44	.21	.58
Q11											.20	.55
Q12												.51

Scale items Q1-Q12 as numbered in Table 1; QT= Q1 through Q12 Total Score.
